# Concept of the five ‘A's for treating emergency arrhythmias

**DOI:** 10.4103/0974-2700.62111

**Published:** 2010

**Authors:** Hans-Joachim Trappe

**Affiliations:** Department of Cardiology and Angiology, University of Bochum, Germany

**Keywords:** Bradycardia, tachycardia, five ‘A’ concept, supraventricular arrhythmias, ventricular arrhythmias, bradyarrhythmias

## Abstract

Cardiac rhythm disturbances such as bradycardia (heart rate < 50/min) and tachycardia (heart rate > 100/min) require rapid therapeutic intervention. The supraventricular tachycardias (SVTs) are sinus tachycardia, atrial tachycardia, AV-nodal reentrant tachycardia, and tachycardia due to accessory pathways. All SVTs are characterized by a ventricular heart rate > 100/min and small QRS complexes (QRS width < 0.12 ms) during the tachycardia. It is essential to evaluate the arrhythmia history, to perform a good physical examination, and to accurately analyze the 12-lead electrocardiogram. A precise diagnosis of the SVT is then possible in more than 90% of patients. In ventricular tachycardia (VT) there are broad QRS complexes (QRS width > 0.12 s). Ventricular flutter and ventricular fibrillation are associated with chaotic electrophysiologic findings. For acute therapy, we will present the new concept of the five ‘A's, which refers to adenosine, adrenaline, ajmaline, amiodarone, and atropine. Additionally, there are the ‘B,’ ‘C,’ and ‘D’ strategies, which refer to beta-blockers, cardioversion, and defibrillation, respectively. The five ‘A’ concept allows a safe and effective antiarrhythmic treatment of all bradycardias, tachycardias, SVTs, VT, ventricular flutter, and ventricular fibrillation, as well as of asystole.

## INTRODUCTION

Emergency medicine and critical care are fields that call for rapid diagnosis and intervention in specific situations.[1] In cardiac emergencies, accurate differentiation of ventricular and supraventricular tachyarrhythmias is essential for appropriate management.[2] Most frequently, the diagnosis of the underlying arrhythmia is readily apparent, but occasionally it is necessary to use clues from the physical examination or the response to maneuvers or drugs, in addition to the 12-lead surface electrocardiogram.[3,4] However, treatment of cardiac arrhythmias in intensive care and emergency medicine is sometimes difficult, although the clinical findings, the physical examination, and the surface electrocardiogram (ECG) will lead to a correct diagnosis in many of them. Correct therapy that is based on an understanding of the mechanism that caused the arrhythmia may not only be lifesaving in the immediate situation but may also improve the quality of life. The purpose of the present manuscript is to present a new concept for the treatment of patients with bradycardia and supraventricular or ventricular tachyarrhythmias in intensive care or cardiac emergencies: the concept of the five ‘A's.

## BRADYARRHYTHMIAS

**Figure 1:**
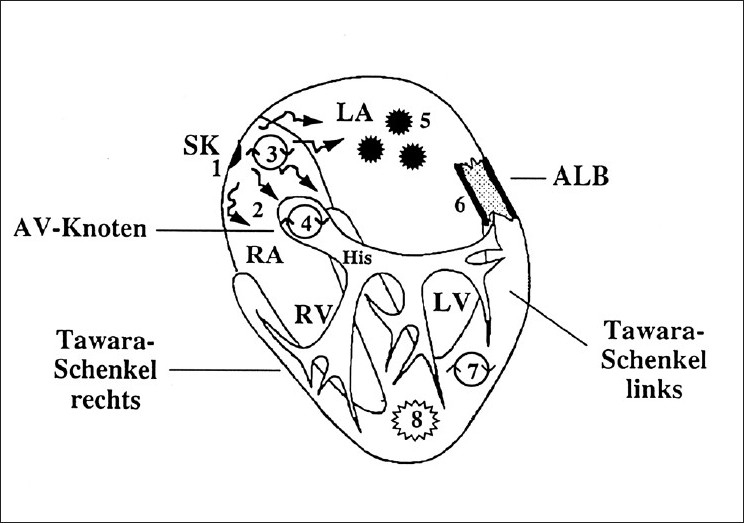
Different forms of supraventricular or ventricular arrhythmias. Abbreviations: 1 = sinus node dysfunction, 2 = sinuatrial conduction disturbances, 3 = atrial conduction disturbances, 4 = AV nodal reentry tachycardia, 5 = atrial tachycardia, 6 = circus movement tachycardia, 7 = ventricular tachycardia, 8 = ventricular flutter/fibrillation

**Figure 2:**
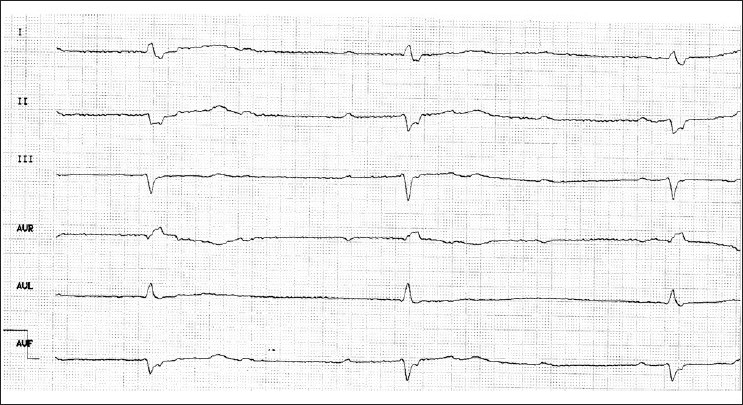
ECG leads I, II, III, aVR, aVL, aVF in a patient with complete AV block

Bradycardia, defined as a heart rate < 50 beats/min, may be caused by different mechanisms and have various etiologies [Table 1]. Sinoatrial (SA) block is a conduction disorder in which impulses generated in the sinus node are intermittently conducted or not conducted to the atrial myocardium. SA conduction abnormalities can be manifest as second-degree block or complete SA block. Second-degree SA block may be type 1 (SA Wenckebach), type 2 (SA Mobitz), or a 2-to-1 SA block (which looks like sinus bradycardia). Sinus arrest is a disorder of automaticity in which no impulses are generated within the sinus node [Figure 1]. Sick sinus syndrome is a dysfunction of the sinus node or SA conduction in which no adequate escape mechanism is present and the patient becomes symptomatic because of the bradycardia. Atrioventricular (AV) block is traditionally divided into first- degree, second-degree, and third-degree blocks. In first-degree AV block, every P wave is conducted to the ventricles but the PR interval is prolonged. Second-degree AV block manifests with P waves that are not conducted to the ventricles. This category is divided into type 1 (Wenckebach), type 2 (Mobitz II), and second-degree AV block with 2-to-1 conduction. Third-degree AV block is characterized by no conduction between the atrium and ventricles [Figure 2]. AV nodal conduction disturbances are seen in acute inferior wall and right ventricular (RV) myocardial infarction when the occlusion is of the right coronary artery above the RV branch. These conduction disturbances can take the form of PR interval prolongation, AV Wenckebach, 2-to-1 block, and complete AV nodal block. Infra-Hisian conduction disturbances are seen in acute anterior myocardial infarction when the occlusion is of the proximal left anterior descending branch; it takes the form of 2-to-1, Mobitz II, or complete block, which are signs of the critical involvement of a large area of the left ventricular myocardium. AV nodal block is common among patients with inferior infarction (incidence 12-20%) and is associated with an increased mortality rate because it usually occurs in the setting of proximal right coronary artery occlusion with RV involvement.[5,6] During the acute phase of such a larg e inferoposterior and RV myocardial infarction due to proximal occlusion of the right coronary artery, approximately 45% of patients have advanced AV nodal block. The incidence of complete AV nodal block in acute inferoposterior wall myocardial infarction is approximately 10%. In patients with anterior myocardial infarction, the incidence of AV nodal block is approximately 5% and it is usually transient. However, in-hospital mortality rates in patients with anterior myocardial infarction and AV nodal block is four times higher compared to those with no conduction disturbances.[7]

**Table 1:** Etiology of bradyarrhythmias

Etiology	Incidence%
Primary	15
Secondary	85
Acute coronary syndrome	40
Toxic	20
Metabolic	5
Neurologic	5
Pacemaker failure	2
Other reasons	13

## NARROW-QRS COMPLEX TACHYCARDIA

Narrow-QRS tachycardia is a cardiac rhythm with a rate faster than 100 beats per minute and a QRS duration of less than 0.12 s. The patient with narrow-QRS tachycardia usually seeks medical attention because of palpitations, light-headedness, shortness of breath, or anxiety. In many patients with narrow-QRS tachycardia, the heart rate is very high (180-240 beats per min) and therefore, after onset of the tachycardia, the patient will arrive very soon in an intensive care unit for diagnosis and treatment.

The narrow-QRS complex indicates that AV conduction occurs through the AV node. ECG documentation of the tachycardia is extremely important so that the mechanism of the tachycardia can be diagnosed and the patient can be given the correct emergency treatment. Causes of narrow QRS tachycardia are sinus tachycardia, atrial tachycardia, atrial flutter, atrial fibrillation, AV nodal reentry tachycardia (AVNRT), and orthodromic circus movement tachycardia (CMT) [Figure 2]. There are many cardiac and extracardiac reasons for supraventricular tachyarrhythmias [Table 2].

**Table 2:** Etiology of tachyarrhythmias

Cardiac reasons
Coronary artery disease
Acute myocardial ischemia
Chronic myocardial ischemia
Cardiomyopathy (dilative, hypertrophic-obstructive, restrictive)
Inflammatory heart disease
Myocarditis
Pericarditis
Congenital heart disease in the adult
Aquired valvular disease
Cardiac neoplasm
Hypertrophy (arterial hypertension)
Long-QT syndrome
Extracardiac reasons
Electrolyte disturbances
Toxic (alcohol)
Liver diseases (hemochromatosis)
Renal diseases
Hypo- and hyperthyroidism
Endocrine diseases
Autoimmune diseases
Neuromuscular diseases (Friedreich ataxia)
Neoplasm
Inflammatory diseases (sarcoidosis, amyloidosis)
Drug-induced reasons
Antiarrhythmics
Digitalis
Psychopharmacological drugs (antidepressives)

## WIDE-QRS COMPLEX TACHYCARDIA

Because a drug given for the treatment of SVT may be deleterious to a patient with ventricular tachycardia (VT), the differential diagnosis in broad-QRS tachycardia is critical. Wide-QRS complex tachycardias (QRS duration > 0.12 s) often pose a difficult diagnostic and therapeutic problem [Figure 1]. Errors are made because emergency care professionals wrongly consider VT unlikely if the patient is hemodynamically stable, and they are often unaware of the ECG findings that quickly and accurately distinguish VT in more than 90% of cases.[8] To make the right diagnosis, it is ideal to have a 12-lead ECG. Diagnostic clues for differentiation of VT from SVT are seen in leads V1 and V6; in addition, a QRS of 0.14 s or more favors a diagnosis of VT. There are several possible mechanisms and reasons of wide- QRS tachycardia [Table 2]. In intensive care and emergencies it is necessary to divide wide-QRS complex tachycardia into those with monomorphic or polymorphic morphologies as well as torsade de pointes tachycardia.[9]

## VENTRICULAR FIBRILLATION AND CARDIAC ARREST

Approximately 1,000 people suffer from cardiac arrest each day in the US, most often as a complication of an acute myocardial infarction with accompanying ventricular fibrillation (VF) or unstable VT [Figure 1]. In 2005, the American Heart Association (AHA) reported again the ‘chain of survival’ concept, with the four links — early access, cardiopulmonary resuscitation, defibrillation, and advanced care — as the way to approach cardiac arrest.[10,11] It has been pointed out that the highest potential survival rate from cardiac arrest can be achieved only when the following sequence of events occurs as rapidly as possible: (a) recognition of early warning signs, (b) activation of the emergency medical services system, (c) institution of basic cardiopulmonary resuscitation, (d) defibrillation, (e) management of the airway and ventilation, and (f) administration of intravenous medications.

## CLINICAL SIGNS IN EMERGENCY DECISION MAKING

In emergencies due to arrhythmias, rapid and correct diagnosis is necessary for adequate therapy. The clinical symptoms, the physical examination, and the 12-lead electrocardiogram are important sources of information for making a correct diagnosis [Table 3]. Of course, it is essential to get information about the underlying heart disease, understand the mechanism of the present arrhythmia, and to decide whether coronary reperfusion is indicated or not. Clinical signs in bradyarrhythmias are hypotension, dizziness, and presyncope or syncope. In patients with the bradycardia–tachycardia syndrome, long pauses are often present in the transition between the tachycardia and the bradycardia, which are caused by inappropriate overdrive suppression of the sinus node. In narrow-QRS complex tachycardia (SVT) careful physical examination during the tachycardia can help establish the origin of the arrhythmia [Table 3]. In all types of regular SVT, the pulse is regular and the blood pressure and loudness of the first heart sound are constant. In atrial fibrillation and atrial flutter with changing AV conduction and changing intervals between successive QRS complexes, the pulse, blood pressure, and loudness of the first heart sound vary. The pulsations of the neck veins often reveal the mechanism of the tachycardia. Rapid regular pulsations (the ‘frog sign’) occur during AVNRT and CMT. In these patients, as a result of simultaneous activation of the atria and ventricles, the atria contract against closed AV valves, producing rapid, regular, expansive venous pulsations in the neck that resemble the rhythmic puffing motion of a frog. Successful termination of the tachycardia by vagal maneuvers suggests AVNRT or CMT by an accessory pathway, whereas syncope suggests rapid SVT, atrial fibrillation with conduction over an accessory pathway, or concomitant structural cardiac abnormalities. In VT, AV dissociation is present in approximately 60% of cases; the other 40% show some form of retrograde conduction to the atria. The finding of AV dissociation is an important diagnostic clue for the diagnosis of VT and it can be diagnosed during the physical examination of the patient: irregular cannon ‘a’ waves in the jugular pulse, variable intensity of the first heart sound, and beat-to-beat changes in the systolic blood pressure are typical signs of AV dissociation [Table 3]. However, in the absence of such clues, VT is still not ruled out.

**Table 3:** Clinical findings and blood pressure behavior in supraventricular tachyarrhythmias

Type of SVA	Pulse	Neck vein	Systolic loudness	Pulsation blood pressure during first heart sound
Sinus tachycardia	Regular	Normal	Constant	Constant
Atrial tachykardia	Regular	Normal	Constant	Constant
AFlut (2:1 con)	Regular	Flutter waves	Constant	Constant
AFlut (irreg con)	Irregular	Irregular	Changing	Changing
AFib	Irregular	Irregular	Changing	Changing
AVNRT	Regular	‘Frog sign’	Constant	Changing
CMT	Regular	‘Frog sign’	Constant	Changing
VT	Regular	Irregular	Changing	Changing

AFIB: ATRIAL FIBRILLATION, AFLUT: ATRIAL FLUTTER, AVNRT: AV NODAL REENTRY TACHYCARDIA, BP: BLOOD PRESSURE, CMT: CIRCUS MOVEMENT TACHYCARDIA, CON: CONDUCTION, HS: HEART SOUND, SVA: SUPRAVENTRICULAR ARRHYTHMIA, VT: VENTRICULAR TACHYCARDIA

In patients with ventricular flutter or ventricular fibrillation, syncope following cardiac arrest is the leading clinical sign.

## THE ECG IN EMERGENCY DECISION MAKING: STEPS FOR CORRECT DIAGNOSIS

**Figure 3:**
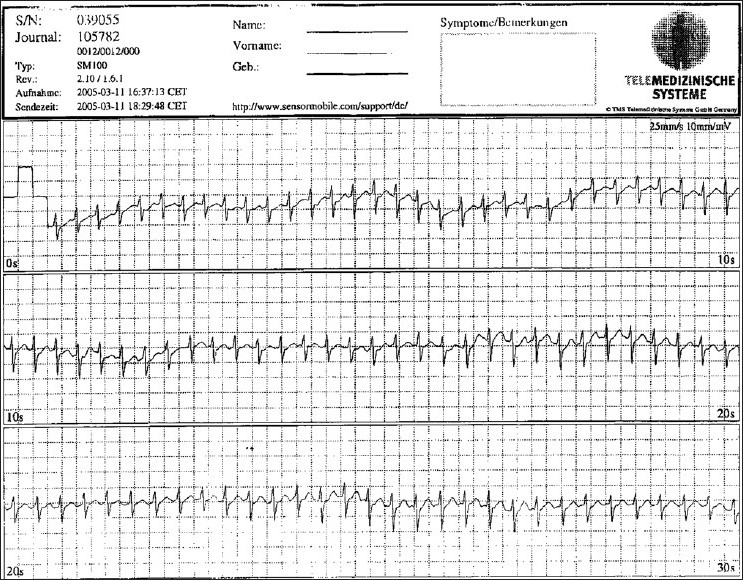
Tele-ECG in a patient with narrow QRS complex tachycardia (QRS width < 0,12 s)

**Figure 4:**
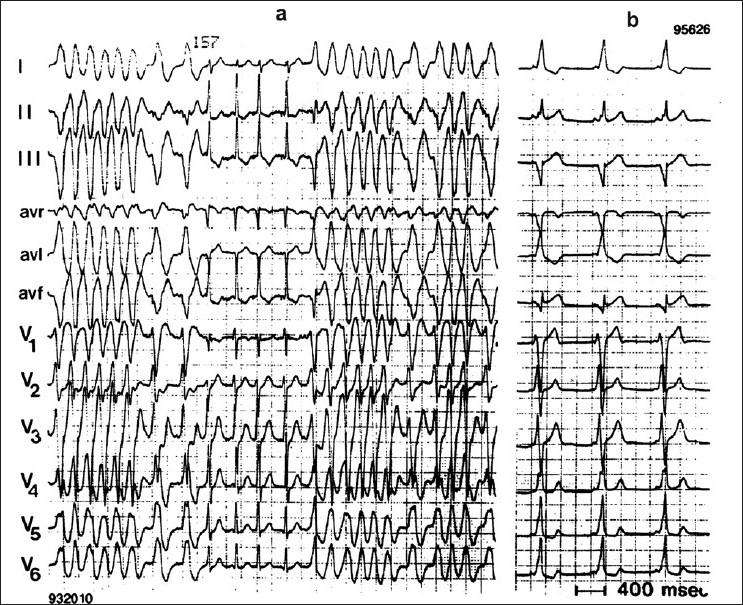
12-lead ECG in a patient with Wolff-Parkinson-White syndrome. Panel a shows atrial fibrillation during preexzitation with rapid ventricular response. Panel b shows the same patient during sinus rhythm

ECG documentation is one of the most important steps for correct diagnosis and treatment. It is necessary to divide tachycardias into those with narrow QRS complexes (QRS < 0.12 s) and those with wide QRS complexes (QRS > 0.12 s) [Figures 3 and 4]. Systematic evaluation of all 12 ECG leads aid in arriving at a correct diagnosis. In addition, it is necessary to understand the mechanisms and ECG features of the different types of narrow-QRS tachycardia [Figure 1]. This has been described in detail elsewhere.[13] The same procedure is necessary in patients with wide-QRS complex tachycardia. Independent atrial and ventricular activity (AV dissociation) during a wide-QRS tachycardia is a hallmark of VT. Other characteristics are signs like right bundle branch or left bundle branch patterns, concordant patterns, Q waves, and QRS onset to S nadir duration. Leads V1 and V6 are important for distinguishing VT from SVT with right bundle branch block morphology.[8]

## CONCEPT OF THE FIVE ‘A'S AS AN EMERGENCY APPROACH IN BRADYARRHYTHMIA AND TACHYARRHYTHMIA

**Figure 5:**
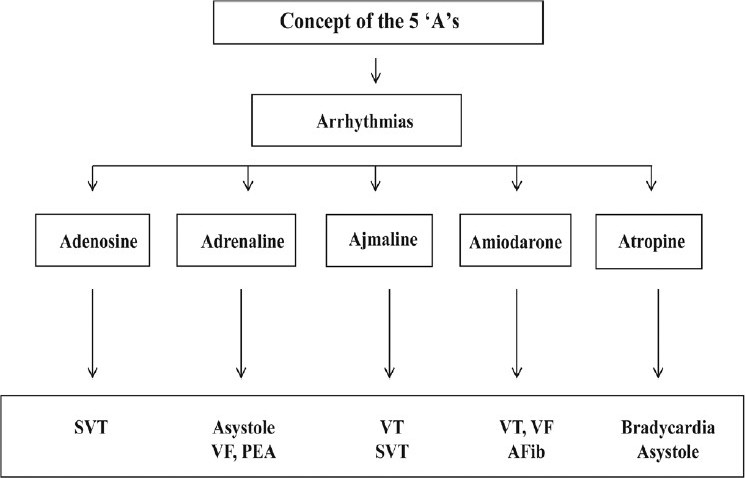
Treatment algorithm for supraventricular and ventricular arrhythmias. Concept of the five “A”s

Sometimes, in emergencies, treatment of bradyarrhythmias, tachyarrhythmias, ventricular flutter, or ventricular fibrillation can be difficult. Younger colleagues and those not familiar with clinical electrophysiology sometimes do not know what to do when a patient develops an arrhythmia. Therefore, we would like to present a new concept for the treatment of arrhythmias in an emergency, a concept that involves only five drugs, all beginning with ‘A’ - adenosine, ajmaline, amiodarone, adrenaline, and atropine - the five ‘A’ concept [Figure 5].

## ADENOSINE

In regular narrow-QRS complex tachycardia, vagal maneuvers should be initiated to terminate the arrhythmia or to modify AV conduction. If this fails, intravenous antiarrhythmic drugs should be administered for arrhythmia termination in hemodynamically stable patients. Adenosine, calcium channel blockers, or beta- blocking agents are the drugs of first choice. Adenosine was approved by the Food and Drug Administration in 1990 and is today the treatment of choice for acute therapy of SVT [Figure 5]. Adenosine is a naturally occurring substance. It binds to cardiac A1 receptors in the SA and AV nodes. Adenosine has negative chronotropic effects in the SA node, depresses conduction, and increases refractoriness in the AV node. Adenosine has a half- life of approximately 10 s. This short half-life is due to rapid metabolism, via deamination, and uptake into cells. The drug does not have clinically significant negative inotropic effects.[12] It is possible to give adenosine in pregnant patients without any risk to the mother or fetus.[14] Adenosine can cause flushing due to peripheral vasodilatation, and it can cause dyspnea due to stimulation of respiration. Some patients may also develop noncardiac chest pain. These symptoms usually resolve within 30-60 s because of adenosine's rapid metabolism. Adenosine is a very effective drug in SVT, with the ability to terminate the arrhythmia in > 90% of cases.[15] It is not effective in terminating atrial flutter or atrial fibrillation, but it does transiently slow the ventricular rate by producing high-grade AV block. In a randomized double-blind trial, adenosine (at a dose of 12 mg) terminated 91% of paroxysmal SVTs compared with 16% with placebo.[16,17] When adenosine (12 mg) was compared with verapamil (12.5 mg) in a randomized double-blind trial, both had very high rates of conversion of paroxysmal SVT to sinus rhythm (93% *vs* 91%, *P* = ns).[16] Adenosine is initially administered as a rapid bolus of 6 mg over 1-2 s. If the SVT is not terminated within 3 min, a 12 mg bolus can be given in a similar fashion. We have demonstrated that SVT termination is possible in 81% of cases when 12 mg adenosine is administered, and it was possible to achieve an even better success rate of 94% with a dose of 18 mg.[18] Higher doses are not recommended as they can result in an increase in sympathetic tone and cause exacerbation of the arrhythmia.

## AJMALINE

The initial approach in patients with wide-QRS complex tachycardia depends on the hemodynamic stability and the symptoms associated with the tachycardia. When the patient is hemodynamically unstable or has pulmonary edema, the tachycardia should be promptly cardioverted with a direct current (DC), synchronized shock. Once the hemodynamically stable patient has been cardioverted and stabilized, it is important to evaluate the preconversion 12-lead ECG for the QRS configuration and signs of AV dissociation as described elsewhere in this manuscript. In patients with sustained (duration > 30 s), hemodynamically stable, monomorphic VT, amiodarone (150-300 mg in 5 min i.v., followed by an infusion of 1050 mg/ day) plays an important role in terminating the arrhythmia; this drug is discussed in detail later. An alternative treatment is the administration of procainamide (10 mg/kg i.v.) or ajmaline (50-100 mg i.v. over 5 min), both of which can provide high termination rates [Figure 5]. In patients with VT in the setting of acute myocardial ischemia, lidocaine (100-150 mg i.v.) was for long the treatment of choice but this therapy is now outdated and is not recommended in the newer guidelines. In addition, it has been well known for a long time that the efficacy of ajmaline is better than that of lidocaine and, besides, lidocaine is associated with a high risk of degeneration of monomorphic VT into ventricular fibrillation.[19] Therefore, lidocaine is no more indicated in patients with VT and should be avoided. When in doubt about the origin of a wide-QRS complex tachycardia, verapamil should not be used and, in these cases, ajmaline is an acceptable drug to terminate both SVT and VT. It has been reported that during emergency treatment of wide-QRS complex tachycardia of unknown origin there was a 44% incidence of severe hemodynamic deterioration when verapamil (5 mg i.v.) was administered during VT, necessitating immediate cardioversion.[20] Hypotension, with resulting ischemia, may render the arrhythmia impossible to cardiovert. Ajmaline (in English-speaking countries the preference is generally for procainamide) has advantages in the termination of both VT and SVT. Ajmaline prolongs the refractory period of the ventricles as well as that of any accessory pathway and the retrograde fast AV nodal pathway. Ajmaline may therefore terminate VT, CMT by an accessory pathway, and the common form of AVNRT [Figure 5].

## AMIODARONE

Amiodarone is currently regarded as the most effective antiarrhythmic drug available for the treatment of patients with both supraventricular and ventricular tachyarrhythmias. It is considered to be particularly useful in patients with life-threatening ventricular tachyarrhythmias [Figure 5]. The most relevant antiarrhythmic effect of amiodarone is due to its prolongation of cardiac repolarization. Amiodarone has been classified as a class-III drug after the Vaughan- Williams classification. However, its low rate of proarrhythmic complications (incidence approximately 1%) as compared to other class-III drugs is at least in part explained by its multiple actions on different ionic channels and ion currents. In patients with sustained (duration > 30 s), hemodynamically stable, monomorphic VT, amiodarone should be administered as bolus initially (150-300 mg in 5 min i.v.), followed by an infusion of 1050 mg/day. In addition, emergency treatment with amiodarone is also clearly indicated in patients with polymorphic VT.[21] Despite all considerations about the ‘ideal’ therapeutic strategy, in patients with polymorphic VT, evaluation of the underlying disease and the mechanism of the arrhythmia is the most important step. In some cases, acute myocardial ischemia is present (‘acute coronary syndrome’) and reperfusion therapy (Percutaneous coronary intervention, thrombolysis, and bypass grafting) will successfully terminate the arrhythmia. Of course, in these cases, reperfusion therapy is indicated and not antiarrhythmic drug treatment. Amiodarone is a highly efficacious antiarrhythmic agent for many cardiac arrhythmias, ranging from atrial fibrillation to malignant ventricular tachyarrhythmias [Figure 5]. In most published studies, intravenous amiodarone has been administered in patients with ventricular tachyarrhythmias only after failure of other antiarrhythmic drugs. In 1999, Kudenchuk studied 504 randomized patients with out-of-hospital cardiac arrest due to refractory ventricular arrhythmias (ARREST study) and reported that treatment with amiodarone (single 300 mg i.v. dose) resulted in a higher rate of survival to hospital admission (44%) compared to placebo (34%) (*P* = 0.03).[21] The role of amiodarone as an emergency drug has been reported recently by Taylor.[22] Today, amiodarone is the drug of choice for patients with VT and in patients with ventricular fibrillation, when DC countershock has failed. Another important indication for amiodarone therapy is in patients with a new atrial fibrillation: amiodarone has a conversion rate in atrial fibrillation of up to 80%.[23,24] Despite many publications on the safety and efficacy of intravenous amiodarone in the treatment of patients with recent-onset atrial fibrillation, there are an increasing number of reports highlighting occasional serious acute pulmonary toxicity in critically ill patients. Therefore, caution in the use of short- term administration of intravenous amiodarone in the critically ill patient with recent-onset atrial fibrillation is absolutely necessary and the duration of therapy should not exceed 24-48 h, except when absolutely necessary

## ADRENALINE

The importance of vital organ perfusion in patients suffering cardiac arrest makes arterial vasomotor tone, and the resultant perfusion pressure, critical in resuscitation from sudden death. After failure of DC countershock, ventilation, and oxygenation, the target organ for resuscitation pharmacotherapy is the arterial vascular smooth muscle cell. There is general agreement that bystander first aid, defibrillation, and advanced life support are essential for good neurologic outcome in patients after a cardiac arrest. Bur *et al*.[25] evaluated the effects of basic life support, time to first defibrillation, and emergency medical service arrival on neurologic outcome in 276 patients after cardiac arrest. In contrast to intubation (odds ratio 1.08; 95% CI: 0.51 to 2.31; *P* = 0.84), basic life support (odds ratio 0.44; 95% CI: 0.24 to 0.77; *P* = 0.004) and time to first defibrillation (odds ratio 1.08; 95% CI: 1.03 to 1.13; *P* = 0.001) were significantly correlated with good neurologic outcome. In addition to the better neurologic outcome, among the patients who did not receive basic life support, the average cost per patient with good neurologic outcome significantly increased with the delay of the first defibrillation (*P* < 0.001). The importance of cerebral perfusion and pressure and cerebral tissue oxygen tension during cardiopulmonary resuscitation has been described elsewhere.[26] Over the last few years the role of pressor drugs in the treatment of cardiac arrest has been discussed extensively. The most commonly used therapy has been catecholamine-induced adrenergic receptor stimulation, with the catecolamine adrenaline (epinephrine) being the commonest drug used. Recently, vasopressin has been the focus of considerable research. Adrenaline is the best studied and most widely administered adrenergic agonist used in the treatment of cardiac arrest. Adrenaline stimulates α_1_ and α_2_ receptors almost equally, and β_1_ and β_2_ receptors in a ratio of approximately 1: 4.[27] The American Heart Association and the European Resuscitation Council continue to recommend repeated administration of adrenaline (with doses of 1 mg i.v.) during advanced cardiopulmonary resuscitation. It has been pointed out that administration of adrenaline is helpful when two countershocks have failed and should be given as soon as possible in patients with asystole or pulseless electrical activity.[10] Few of the early investigations of adrenaline in cardiac arrest addressed the issue of dosage, with many studies using a single dose independent of body weight. In 1906, Crile and Dolley gave ‘1-2 cc (ml) of 1:1000 solution of adrenaline’ to dogs.[28] This solution was adrenaline chloride, and the dose has been estimated to have been comparable to approximately 0.4 mg/kg. Many years later, the role of ‘high-dosage’ adrenaline strategies was discussed extensively and early human studies of high-dose adrenaline, which tended to be performed in patients who had failed conventional doses, reported improvements in variables such as coronary perfusion pressure and the rate of ‘return of spontaneous circulation’. The first large well-designed clinical trial of high-dose adrenaline was performed by Brown *et al*.[29] They compared 0.02 mg/kg adrenaline with 0.2 mg/kg adrenaline in 1280 patients suffering out-of-hospital cardiac arrest. However, this study found no significant difference in clinical outcome between the two g roups. Today, high-dose therapy with adrenaline does not play any role in the treatment of patients with out-of-hospital cardiac arrest.

Another important vasopressor drug is vasopressin. It acts directly on contractile elements by way of V_1_ receptors. Wenzel *et al*.[30] studied vasopressin (40 units i.v.) in 1219 patients with out-of-hospital cardiac arrest. These patients were randomized into a vasopressin group (40 units i.v.) and an adrenaline group (1 mg i.v.). There were no significant differences between vasopressin and adrenaline in hospital admission when ventricular fibrillation (46.2% *vs* 43.0%) or pulseless electrical activity were present (33.7% *vs* 30.5%) (*P* = ns). In accordance with these results, the guidelines of the American Heart Association and European Resuscitation Council recommended 40 units vasopressin intravenously and 1 mg adrenaline intravenously as equally effective for the treatment of patients with ventricular fibrillation [Figure 5]. There is no evidence to show that any one of these drugs is superior to the other.[30]

## ATROPINE

Atropine is a tropane alkaloid extracted from deadly nightshade (*Atropa belladonna*), jimsonweed (*Datura stramonium*), mandrake (*Mandragora officinarum*), and other plants of the family Solanaceae. It is a secondary metabolite of these plants and serves as a drug with a wide variety of effects. It is a competitive antagonist for the muscarinic acetylcholine receptor. It is classified as an anticholinergic drug. Atropine increases firing of the SA node and conduction through the AV node, opposes the actions of the vagus nerve, blocks acetylcholine receptors, and decreases bronchiole secretions. In general, atropine lowers the parasympathetic activity of all muscles and glands regulated by the parasympathetic nervous system. This occurs because atropine is a competitive antagonist of the muscarinic acetylcholine receptors. Acetylcholine is the main neurotransmitter used by the parasympathetic nervous system. Atropine may cause swallowing difficulties and reduced secretions. Atropine works because the main action of the vagus nerve of the parasympathetic system on the heart is to decrease the heart rate. Atropine blocks this action and, therefore, may increase the heart rate. Indications for atropine administration are vagus-mediated sinus bradycardia, blocks in the AV node, and vagus-mediated asystole.[10] For symptomatic bradycardia or when there is asystole, the usual dosage of atropine is 0.5-1.0 mg i.v. every 3-5 min, up to a maximum dose of 0.04 mg/kg (3 mg) [Figure 5]. Atropine is also useful in treating second-degree AV block (Mobitz block) and in third-degree heart block with a high Purkinje or AV-nodal escape rhythm. It is usually not effective in third-degree heart block with a low Purkinje or ventricular escape rhythm. Atropine is contraindicated in ischemia-induced conduction block because the drug increases the oxygen demand of the AV nodal tissue, thereby aggravating the ischemia and the resulting heart block.In addition, atropine is not indicated in patients with bradycardia due to infranodal second-degree AV block. In this situation, a paradoxic bradycardia can occur with atropine.[31]

## CONCLUSIONS

Emergency medicine and critical care are fields that often require rapid diagnosis and intervention for specific situations. It is well known that in all patients with tachyarrhythmias, evaluation of the underlying etiology and the degree of left ventricular dysfunction is essential. Correct treatment of arrhythmias in the intensive care setting is based on an understanding of the mechanisms that cause the situation. The therapeutic role of antiarrhythmic drugs in the management of atrial fibrillation or cardiac arrest is debatable. For acute therapy, there is the new concept of the five ‘A's, which refers to adenosine, adrenaline, ajmaline, amiodarone, and atropine. The five ‘A's concept will enable safe and effective treatment of all bradycardias, tachycardias, SVTs, VT, ventricular flutter, ventricular fibrillation, and of asystole.
